# Biotechnological Transformation of Hydrocortisone into 16α-Hydroxyprednisolone by Coupling *Arthrobacter simplex* and *Streptomyces roseochromogenes*

**DOI:** 10.3390/molecules25214912

**Published:** 2020-10-23

**Authors:** Odile Francesca Restaino, Simona Barbuto Ferraiuolo, Addolorata Perna, Marcella Cammarota, Maria Giovanna Borzacchiello, Antonio Fiorentino, Chiara Schiraldi

**Affiliations:** 1 Department of Experimental Medicine, Section of Biotechnology and Molecular Biology, University of Campania “Luigi Vanvitelli”, Via De Crecchio 7, 80138 Naples, Italy; simona.barbutoferraiuolo@unicampania.it (S.B.F.); dolores.90@libero.it (A.P.); marcella.cammarota@unicampania.it (M.C.); mariagiovanna.borzacchiello@unicampania.it (M.G.B.); chiara.schiraldi@unicampania.it (C.S.); 2 Department of Environmental, Biological and Pharmaceutical Sciences and Technologies, University of Campania “Luigi Vanvitelli”, Via Vivaldi 43, 81100 Caserta, Italy; antonio.fiorentino@unicampania.it

**Keywords:** *Arthrobacter simplex*, bioconversion, hydrocortisone, 16α-hydroxyhydrocortisone, 16α-hydroxyprednisolone, prednisolone, *Streptomyces roseochromogenes*

## Abstract

16α-Hydroxyprednisolone, an anti-inflammatory drug, could be potentially obtained from hydrocortisone bioconversion by combining a 1,2-dehydrogenation reaction performed by *Arthrobacter simplex* *ATCC*31652 with a 16α-hydroxylation reaction by *Streptomyces roseochromogenes ATCC*13400. In this study we tested, for the first time, potential approaches to couple the two reactions using similar pH and temperature conditions for hydrocortisone bioconversion by the two strains. The *A. simplex* capability to 1,2-dehydrogenate the 16α-hydroxyhydrocortisone, the product of *S. roseochromogenes* transformation of hydrocortisone, and vice versa the capability of *S. roseochromogenes* to 16α-hydroxylate the prednisolone were assessed. Bioconversions were studied in shake flasks and strain morphology changes were observed by SEM. Whole cell experiments were set up to perform the two reactions in a sequential mode in alternate order or contemporarily at diverse temperature conditions. *A. simplex* catalyzed either the dehydrogenation of hydrocortisone into prednisolone efficiently or of 16α-hydroxyhydrocortisone into 16α-hydroxyprednisolone in 24 h (up to 93.9%). Surprisingly *S. roseochromogenes* partially converted prednisolone back to hydrocortisone. A 68.8% maximum of 16α-hydroxyprednisolone was obtained in 120-h bioconversion by coupling whole cells of the two strains at pH 6.0 and 26 °C. High bioconversion of hydrocortisone into 16α-hydroxyprednisolone was obtained for the first time by coupling *A. simplex* and *S. roseochromogenes*.

## 1. Introduction

**Figure 1:**
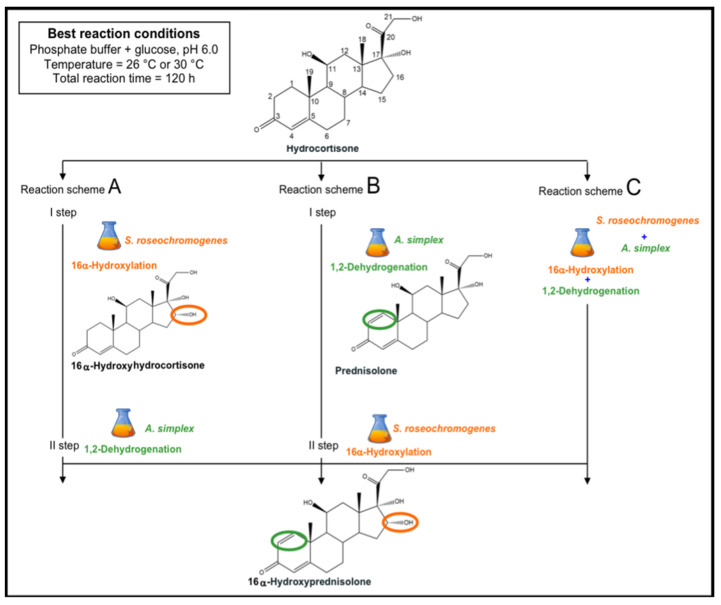
Possible reaction scheme to obtain 16α-hydroxyprednisolone from hydrocortisone transformation by coupling the 1,2-dehydrogenation by *A. simplex* with the 16α-hydroxylation by *S. roseochromogenes*: the two reactions might potentially be performed in a sequential mode in different order (**A**,**B**) or at the same time (**C**).

Microbial transformations are widely used to obtain steroidal drugs with high regio- and stereo-specificity [1,2,3,4,5]. *Corynebacterium, Nocardia, Mycobacterium, Rhizopus*, *Aspergillus*, *Streptomyces, Arthrobacter*, *Penicillium* and *Rhodococcus* genera have been demonstrated to be able to perform reactions such as hydroxylation, oxidation, dehydrogenation or halogenation of steroid compounds including estrane, pregnane and androstane at specific positions of the rings with high selectivity [6,7]. Both hydroxylation and dehydrogenation reactions performed by whole microbial cells or their enzymes, as biocatalysts, have been widely used to modify steroidal drugs to obtain higher pharmaceutical efficacy [7]. *Streptomyces* species (e.g., *Streptomyces argenteolus* and *Streptomyces roseochromogenes*) are able to convert different substrates such as testosterone, 1-dehydrotestolactone, pregnenolone, progesterone and dehydroepiandrosterone into their 16α-hydroxylated derivates, with high specificity thanks to a cytochrome P450 multi enzymatic complex. Thus, these species could be employed to obtain new efficient anti-inflammatory agents with higher glucocorticoid activities [3,8]. *Arthrobacter* strains (e.g., *Arthrobacter simplex* and *Arthrobacter globiformis*) have been widely used to introduce a 1,2 double bond in corticoid rings thanks to a Δ^1^-dehydrogenase, a structural change that enhances the affinity for the glucocorticoid receptors and increases the therapeutic activity of steroidal drugs [3,9]. In the literature, for example, the Δ^1^-dehydrogenation of 6-methylcortisol into 6-methylprednisolone by using *Arthrobacter globiformis* whole cells was explored [3,10]. The transformation of cortisone acetate into prednisone acetate by *Arthrobacter simplex* whole or immobilized cells has also been widely described [11,12,13]. 16α-hydroxyprednisolone (16α-OH-PD) or desfluorotriamcinolone is a highly effective anti-inflammatory drug that could potentially be obtained through the biotechnological bioconversion (BC) of hydrocortisone (HC) by coupling a 1,2 -dehydrogenation reaction with a 16α-hydroxylation [14]. Two recent studies have demonstrated the possibility to employ *Streptomyces roseochromogenes* as a whole cell biocatalyst to perform the first step of bioconversion from hydrocortisone to 16α-hydroxyhydrocortisone (16α-OH-HC) [14,15]. In those studies, the growth and bioconversion capability of *S. roseochromogenes* were first investigated on two new-formulated media, which contained glucose, yeast extract and ammonium sulfate (GYA) or glucose, yeast extract and malt extract (GEM III N) [14,15]. Different culture conditions were exploited but the strain was demonstrated to highly convert HC in 16α-OH-HC only when grown on GEM III N medium at pH 6.0 and 26 °C; in these conditions, up to 80.0% of conversion was reached in 96 h, with a low formation (<10.0%) of by-products, for example 20-hydroxyhydrocortisone (20-OH-HC) [14,15]. The 1,2-dehydrogenation reaction of hydrocortisone to obtain prednisolone (PD), as a precursor of 16α-hydroxyprednisolone, performed by whole cells of *A. simplex*, has been poorly investigated so far. Furthermore, the possibility to couple *A. simplex* and *S. roseochromogenes* to perform in a sequential mode or at the same time the 1,2-dehydrogenation reaction and the 16α-hydroxylation of hydrocortisone to obtain the 16α-hydroxyprednisolone has not yet been reported in literature. In fact, papers describing the use of *Arthrobacter* and/or *Streptomyces* strains in multistep steroid bioconversions, even in combination with other strains, to perform dehydrogenation and hydroxylation reactions, are very few [3,7]. Immobilized *A. simplex* cells, for example, were used in combination with immobilized *Curvularia lunata* mycelia to transform cortexolone into prednisolone in a two-step bioconversion including a Δ^1^-dehydrogenation and a 11β-hydroxylation reaction [16]. In only one proceeding of a meeting, the two strains were coupled together in a mixed co-culture to study the kinetic parameters of the conversion of 9α-fluorohydroxycortisone into Δ^1^-dehydro-9α-fluoro-16α-hydroxyhydroxycortisone [17]. In this study, for the first time, we tried to obtain 16α-hydroxyprednisolone by coupling the transformation capabilities of *A. simplex* and *S. roseochromogenes*. In this perspective, *A. simplex* shake flask experiments were performed first by investigating diverse growth media, temperature and pH values. These experiments were set up to find the culture and conversion conditions of hydrocortisone into prednisolone similar to those previously used for *S. roseochromogenes* biotransformation. Further studies were then performed to investigate the ability of *A. simplex* to also catalyze 1,2-dehydrogenation of the 16α-hydroxyhydrocortisone obtained by *S. roseochromogenes* transformation of HC and vice versa if *S. roseochromogenes* could 16α-hydroxylate the prednisolone obtained by *A. simplex* bioconversion of HC (Figure 1). Eventual changes of the morphology of the two strains during the bioconversion experiments were also investigated by SEM. Then, 20-mL scale systems were set up to obtain 16α-hydroxyprednisolone by using whole cells of the two microorganisms, starting from 0.1 g·L^−1^ of hydrocortisone. The 1,2-dehydrogenation reaction by *A. simplex* and the 16α-hydroxylation reaction by *S. roseochromogenes* were performed in a sequential mode in alternate order or at the same time according to three different reaction schemes (Figure 1).

## 2. Results

### 2.1. A. simplex Shake Flask Experiments

**Figure 2:**
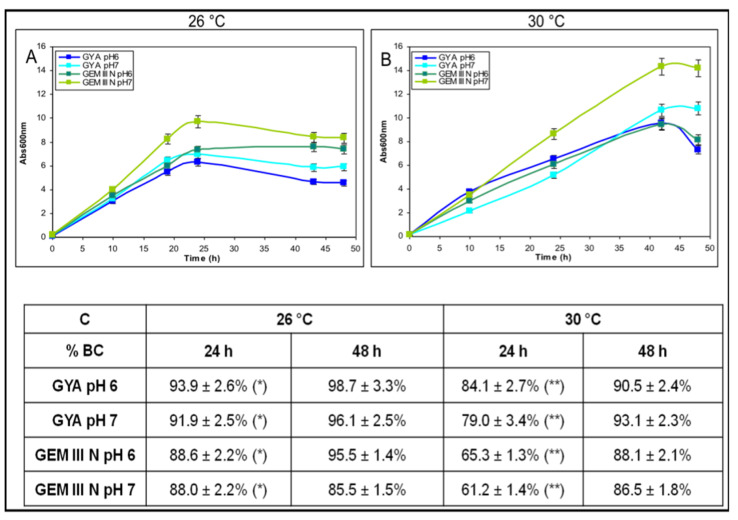
Shake flask experiments of *A. simplex*: growth on GYA or GEM III N media, at pH 6.0 or 7.0, at 26 °C (**A**) or at 30 °C (**B**) and the correspondent bioconversion percentages of 0.1 g·L^−1^ HC to prednisolone (**C**). HC was added at time zero (* indicates *p* < 0.05; ** indicates *p* < 0.01).

*A. simplex* physiological studies were performed in shake flasks on two different media at two diverse pH and temperature values in order to test the effect of various conditions on both the microbial growth and the bioconversion of HC into prednisolone (Figure 2). Comparing the growth curves at 26 °C it was noted that the maximum biomass values were reached at 24 h (values from 6.3 ± 0.2 to 9.7 ± 0.3 Abs600 nm) and that *A. simplex* grew better on GEM III N and at pH 7.0 than in the other conditions (Figure 2A). At 30 °C, the bacteria grew more and for longer as the maximum biomasses were reached at 42 h. Their values were 2.2 times higher than the ones reached at 26 °C (from 9.4 ± 0.4 to 14.2 ± 0.5 Abs600 nm), and the best growth was obtained on GEM III N at pH 7.0 (Figure 2B). At 30 °C, almost all the initial glucose concentration (80.0–89.0%) was consumed while at 26 °C the total glucose consumption was about 68.0–72.0% of the initial amount (Table S1). The study of the HC kinetic of bioconversion into prednisolone showed that, differently from the growth, the transformation worked better on GYA than on GEM III N and slightly more quickly at 26 °C than at 30 °C independently of the pH used (Figure 2C). At 26 °C on GYA medium, the conversion values were between 93.9% and 91.9% at 24 h and between 98.7% and 96.1% at 48 h, at pH 6.0 and 7.0, respectively (Figure 2C). Slightly lower values of bioconversion were obtained at 30 °C: between 84.1% and 79.0% at 24 h and between 90.5% and 93.1% at 48 h, at pH 6.0 and 7.0, respectively (Figure 2C). On GEM III N medium at 26 °C, the bioconversion values of HC were around 88.0% at 24 h at both pH values and between 65.3% and 61.2% at 30 °C, at pH 6.0 and 7.0, respectively. Higher values, from 85.5% to 95.5%, were noted at 48 h. Statistical analyses demonstrated that the differences between the 24 h bioconversion obtained at 26 °C on GYA medium at pH 6.0 and 7.0 were significantly higher than the ones obtained on GEM III N medium at pH 6.0 and 7.0 at the same temperature and time point (*p* < 0.05). These differences were even more significant at 30 °C (*p* < 0.01) (Figure 2C).

### 2.2. Shake Flask Experiments on Different Substrates

**Figure 3:**
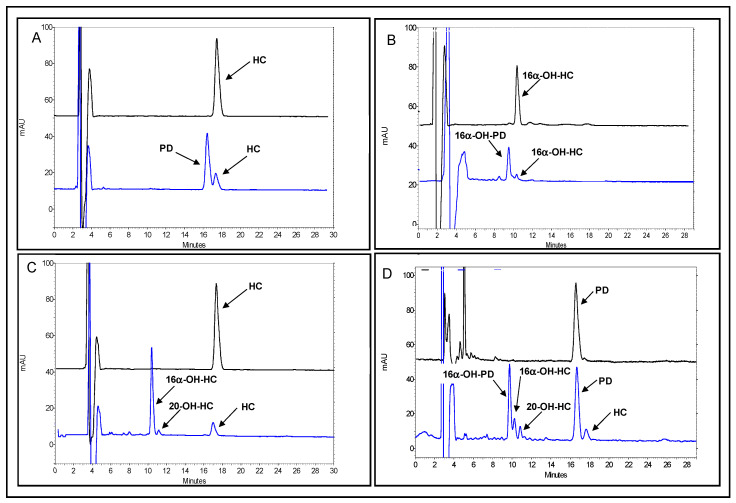
Representative HPLC chromatograms of *A. simplex* dehydrogenation of hydrocortisone into prednisolone (**A**) and of 16α-hydroxyhydrocortisone into 16α-hydroxyprednisolone (**B**) and of *S. roseochromogenes* hydroxylation of hydrocortisone into 16α-hydroxyhydrocortisone (and into the by-product, 20-hydroxyhydrocortisone) (**C**) and of prednisolone into 16α-hydroxyprednisolone (**D**). In this last case, the microorganism also performed a hydrogenation reaction of prednisolone back to hydrocortisone that was then hydroxylated into 16α-hydroxyhydrocortisone (and into the by-product, 20-hydroxyhydrocortisone) (**D**). All peaks are indicated by the arrows.

**Figure 4:**
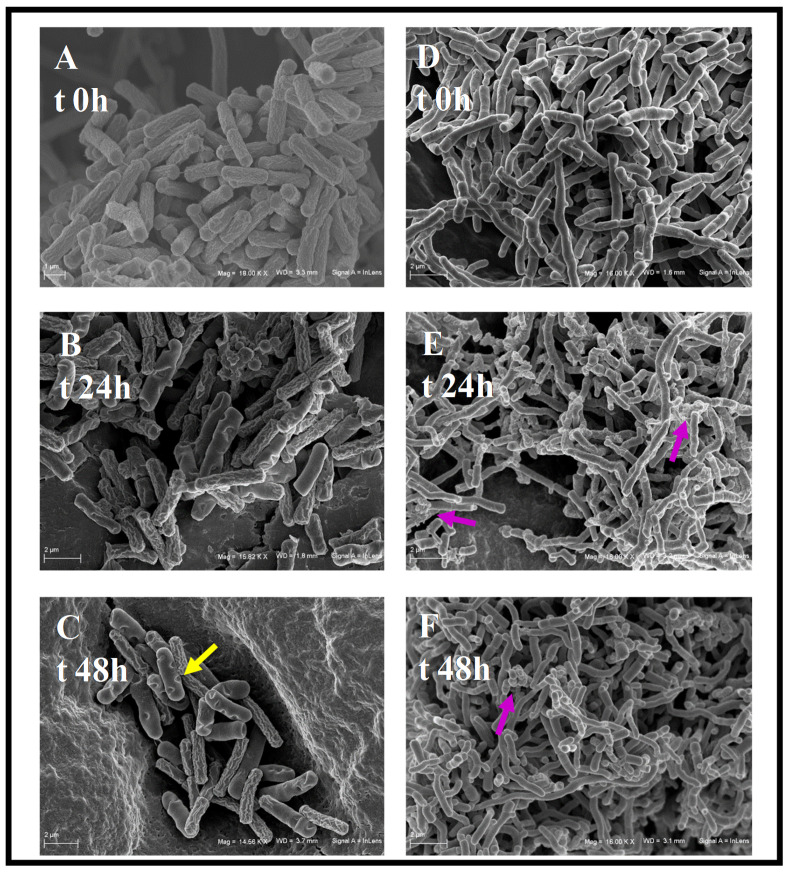
Changes of cell morphology at different time points of the growths and of the bioconversion processes of *A. simplex* (**A**–**C**) and *S. roseochromogenes* cells (**D**–**F**) detected by scanning electron microscopy (~16,000, scale bar 1 or 2 µm). *A. simplex* introflections and concave notches are indicated by the yellow arrow, while the accumulation of material on *S. roseochromogenes* cell surface is indicated by purple arrows.

The study of the bioconversion reactions of the two strains not only on HC but also on the bioconversion product of the other microorganism transformation is also a key point in the perspective to couple *A. simplex* with *S. roseochromogenes* in whole cell experiments in order to obtain 16α-hydroxyprednisolone. In fact, 16α-hydroxyprednisolone can only be obtained from hydrocortisone if *S. roseochromogenes* can also hydroxylate prednisolone and/or if *A. simplex* can also dehydrogenate 16α-hydroxyhydrocortisone (Figure 1). To solve this issue new *A. simplex* and *S. roseochromogenes* shake flask studies were performed by using 16α-hydroxyhydrocortisone and prednisolone as substrates, respectively. Data were compared with the ones obtained when hydrocortisone was used as starting material. *A. simplex* was able to convert 16α-hydroxyhydrocortisone similarly to hydrocortisone and 92.0 ± 1.8% 16α-hydroxyhydroprednisolone was obtained in 24 h (Figure 3A,B). In 96-h runs, *S. roseochromogenes* was able to hydroxylate the prednisolone into 16α-hydroxyprednisolone (24.1 ± 2.4%) but in this case the microorganism also performed a hydrogenation reaction, transforming prednisolone back to hydrocortisone (13.9 ± 1.5%) that was then further hydroxylated into 16α-hydroxyhydrocortisone (18.2 ± 2.2%) as well as into its side product, 20-hydroxyhydrocortisone (10.5 ± 1.7%) (Figure 3C,D). When hydrocortisone was used as substrate, *S. roseochromogenes* only performed the bioconversion into 16α-hydroxyhydrocortisone (82.4 ± 1.6%) and into its side product, 20-hydroxyhydrocortisone (4.5 ± 1.4%) (Figure 3C,D), similar to what has already been reported [14,15]. *A. simplex* and *S. roseochromogenes* growth curves, obtained when 16α-hydroxyhydrocortisone and prednisolone were used as substrates, respectively, were similar to the curves reported above and before when HC was used as substrate, with maximum biomass values of 6.5 ± 0.3 and 8.1 ± 0.1 Abs600 nm, respectively (Figure S1) [14,15]. The morphology changes of the two strains during the bioconversion processes were also studied and analyzed by SEM (Figure 4). *A. simplex* showed a rod shape and an extensive wrinkling of the outer membrane at the beginning of the growth, but the rugosity of the cell surface slightly decreased between 18 and 24 h (Figure 4A–C). At the end of the growth, some introflections and concave notches were found on the cell wall and a partial loss of the initial roughness was also detectable (yellow arrow in Figure 4C). *S. roseochromogenes* morphology changed during the growth as well: initially an increase of the population was noted and signs of budding due to cell duplication were clearly visible (Figure 4D–F). Starting from 24 h, accumulation of material on the cell surface was clearly visible, probably due to the process of steroid bioconversion, and from 48 h increases of cell tangling, branching and rugosity were also noted (purple arrows in Figure 4E,F).

### 2.3. Whole Cell Experiments

#### 2.3.1. Whole Cell Experiments by Using *S. roseochromogenes* and *A. simplex* in a Sequential Mode

**Figure 5:**
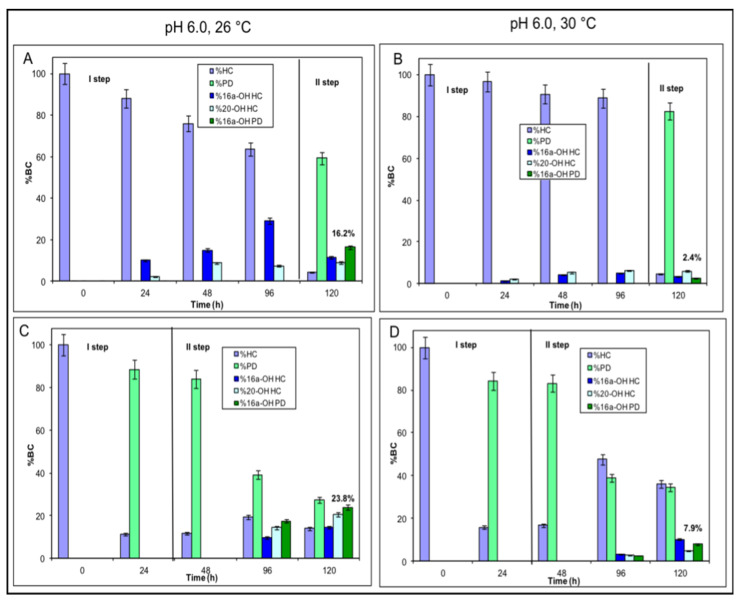
Bioconversion of 0.1 g·L^−1^ HC into different products in whole cell experiments at pH 6.0 and 26 or at 30 °C obtained by employing *S. roseochromogenes* first and then *A. simplex*, respectively (**A**,**B**) or *A. simplex* first, and then *S. roseochromogenes*, respectively (**C**,**D**). In the graphs, a black line separates the two steps of the experiments. The 16α-OH-prednisolone percentages obtained are reported in bold.

Aiming to obtain 16α-hydroxyprednisolone by bioconversion, 20-mL scale systems were set up by using whole cells of the two microorganisms. In a first set of experiments, *S. roseochromogenes* and then *A. simplex* were used in a sequential mode to bioconvert 0.1 g∙L^−1^ HC at pH 6.0 and at 26 or 30 °C (Figure 1, Reaction Scheme A). In both temperature conditions, *S. roseochromogenes* partially used the substrate to perform the hydroxylation reactions and a maximum value of 29.1 ± 0.4% of 16α-OH-HC was obtained at 26 °C in 96 h (Step I in Figure 5A,B). The strain also produced the 20-OH-HC by-product, up to 7.3 ± 0.3%. When the supernatants of the *S. roseochromogenes* bioconversion reactions were used by *A. simplex* cells the residual HC was quickly converted to prednisolone in 24 h (Step II in Figure 5A,B). In the experiments performed at 30 °C, the HC not converted by *S. roseochromogenes* was higher and thus a higher percentage of PD was obtained by bioconversion of *A. simplex* (88.8 ± 0.2% of the remaining HC was converted into 82.5 ± 0.5% of PD) (Step II in Figure 5B). In both temperature conditions, *A. simplex* also dehydrogenated the 16α-OH-HC produced by *S. roseochromogenes* into 16α-OH-PD. As the highest percentage of 16α-OH-HC was obtained by *S. roseochromogenes* at pH 6.0 and 26 °C, the highest percentage of 16α-OH-PD (16.2 ± 0.5%) was obtained at these conditions (the sum of the 16α-OH-HC and 16α-OH-PD percentages at 120 h is equivalent to the percentage of 16α-OH-HC at 96 h) (Step II in Figure 5A). The lower percentages of 16α-OH-prednisolone obtained at pH 6.0 and 30 °C were clearly due to the lower bioconversion of 16α-OH-HC reached during the first step of bioconversion by using *S. roseochromogenes* (Step II in Figure 5B).

#### 2.3.2. Whole Cell Experiments by Using *A. simplex* and *S. roseochromogenes* in a Sequential Mode

In a second set of experiments *A. simplex* whole cells and then *S. roseochromogenes* cells were employed in a sequential mode (Figure 1, Reaction Scheme B) to convert 0.1 g∙L^−1^ HC at two different incubation temperatures (26 and 30 °C) and at pH 6.0. *A. simplex* demonstrated to quickly convert HC into PD at both 26 and 30 °C reaching values of 88.6 ± 0.6% and 84.2 ± 0.2% in 24 h, respectively (Step I in Figure 5C,D). When the supernatants of these two bioconversions were used as substrates for *S. roseochromogenes*, it was noted that the strain was able to convert the prednisolone into 16α-OH-prednisolone in 96 h with percentages higher at 26 °C than at 30 °C (BC of 23.8 ± 0.7% and 7.9 ± 0.6%, respectively) (Step II in Figure 5C,D). The strain also demonstrated once again to be able to partially hydrogenate the prednisolone back to HC. The 11.4% and 15.8% of HC that was not converted by *A. simplex* increased up to 19.4 ± 0.4% and 47.6 ± 0.3% in 72 h of *S. roseochromogenes* bioconversion, as the hydrogenation reaction seemed to work better at 30 °C than at 26 °C. The produced HC was then further converted into the two hydroxylated forms of 16α-OH-HC (up to 14.4 ± 0.4% and 10.2 ± 0.8% at 26 and 30 °C, respectively, at 120 h) and of 20-OH-HC (20.5 ± 0.6% and 4.9 ± 0.3% at 26 and 30 °C, respectively). As always in the case of a hydroxylation reaction, the strain worked better at 26 °C than at 30 °C (Step II in Figure 5C,D). In its 24-h step, *A. simplex* consumed 64.3 ± 1.6% and 83.4 ± 1.7% of the initial glucose amount at 26 and 30 °C, respectively, while, in its 96-h step, *S. roseochromogenes* consumed 98.2 ± 1.0% and 89.4 ± 0.8% of the initial glucose amount at 26 and 30 °C respectively.

#### 2.3.3. Whole Cell Experiments Coupling *A. simplex* and *S. roseochromogenes*

**Figure 6:**
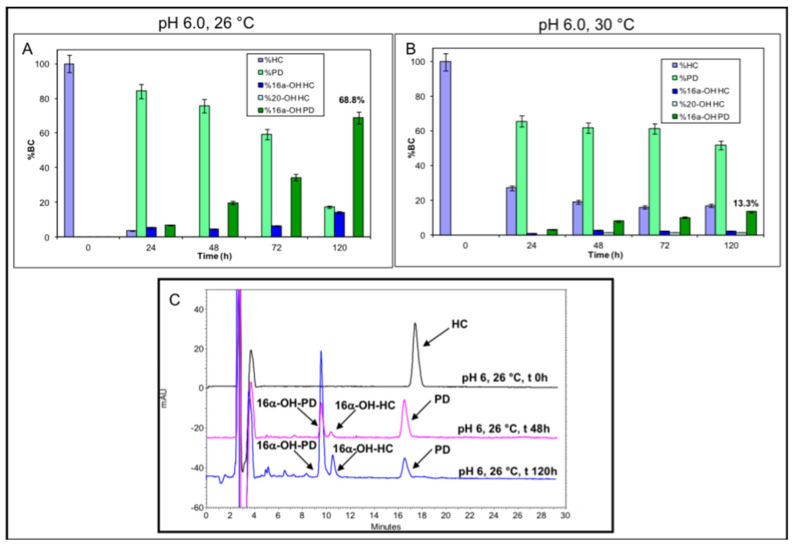
Bioconversion of 0.1 g·L^−1^ HC into different products in whole cell experiments at pH 6.0 and at 26 or 30 °C, obtained by coupling *A. simplex* and *S. roseochromogenes*, respectively (**A**,**B**). In the graphs, the obtained 16α-OH-prednisolone percentages are reported in bold. Overlaid HPLC chromatograms of the 0.1 g·L^−1^ HC bioconversion samples at pH 6.0 and 26 °C at different time points (**C**) (0 h, black line; 48 h, pink line; 120 h, blue line). All peaks are indicated by arrows.

In a third set of experiments, *A. simplex* and *S. roseochromogenes* whole cells were coupled together to convert 0.1 g∙L^−1^ HC at pH 6.0 and at 26 or 30 °C. *A. simplex* dehydrogenation of HC to prednisolone was quicker than the hydroxylation reaction performed by *S. roseochromogenes*. In 24 h, 84.2 ± 0.2% and 65.6 ± 0.4% of bioconversion were obtained at 26 and 30 °C, respectively (Figure 6A,B). In these coupled experiments, the *S. roseochromogenes* hydroxylation ability seemed to be influenced by both temperature conditions and the concentration of prednisolone produced by *A. simplex,* as already seen in sequential mode experiments. At 26 °C, a maximum of 68.8 ± 0.2% of 16α-OH-prednisolone was obtained at 120 h and a residue of prednisolone was present (17.3 ± 0.7%); 13.9 ± 0.4% of 16α-OH-HC was also produced (Figure 6C). The hydroxylation reaction had the lowest efficiency at 30 °C and only 13.3 ± 0.5% of 16α-OH-prednisolone was obtained. Lower percentages of 16α-OH-HC (2.5 ± 0.2%) and 20-OH-HC (1.4 ± 0.4%) were observed (Figure 6A,B). At the end of the experiments, glucose consumption was 95.5 ± 0.5% at 26 °C and 98.2 ± 0.8% at 30 °C.

### 2.4. 16α-OH-PD Purification and Characterization

**Figure 7:**
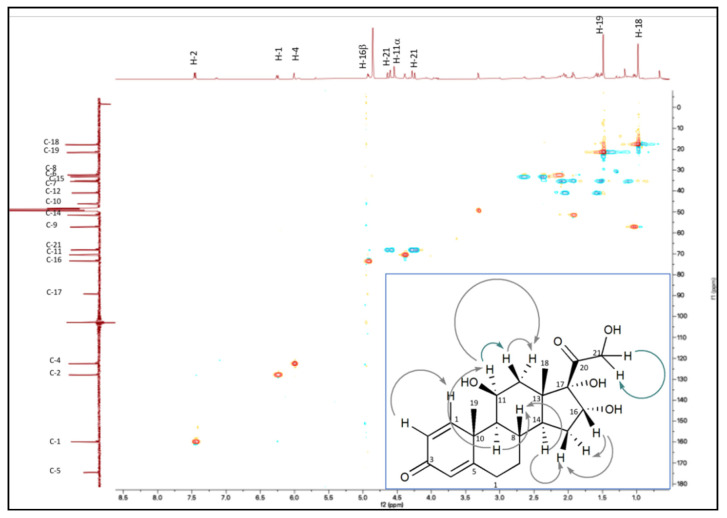
Selected HSQC correlations of 16α-OH prednisolone obtained by bioconversion of HC by coupling *A. simplex* and *S. roseochromogenes.* In the figure COSY correlations are shown.

The peak of 16α-OH-PD obtained in the bioconversion experiments was identified by HPLC analyses on the base of the elution time by comparison with the 16α-OH-PD standard (Figure S2A). The 16α-OH-PD structure was also double checked by performing one- and two-dimensional NMR experiments after chromatographic purification. To this aim, three 20-mL whole cell experiments were performed at pH 6.0 and 26 °C to convert 0.1 g∙L^−1^ HC by coupling *A. simplex* and *S. roseochromogenes*, as described above. After 120 h of bioconversion, the supernatants were collected by centrifugation and then loaded to a preparative chromatographic column to purify the obtained 16α-OH-prednisolone. HPLC analyses determined a 92.0% of purity of the product obtained by preparative chromatography (Figure S2B). The structure of the purified 16α-OH-prednisolone was controlled by ^1^H, ^13^C, DQF-COSY and HSQC NMR (Table 1 and Supplementary Figures S3–S5). The ^1^H NMR spectrum of the purified bioconversion product showed the H-18 and H-19 methyl signals as two singlets at δ 0.98 and 1.48, respectively. The ^13^C NMR showed the presence of 21 carbon signals, identified, on the basis of a HSQC experiment (Figure 7), as two methyls, six methylenes, seven methines and six quaternary carbons. The presence of the *α*,*β*-double-unsaturated ketone of the ring A in the ^1^H-NMR spectrum was confirmed by two doublets at δ 7.45 (H-1, J = 10.0 Hz) and 6.25 (H-2, J = 10.0 Hz) and the H-4 singlet at δ 6.01. In the region of the protons germinal to oxygen, the AB system of the H-21 methylene was present as two doublets at δ 4.62 (J =19.5 Hz) and 4.26 (J = 19.5 Hz), besides the H-11 proton as a broad singlet at δ 4.39. The presence of a doublet at δ 4.92 (J = 8.5 Hz) suggested the presence of a further hydroxyl group in the molecule. This latter proton, correlated in the HSQC experiment to a carbinol carbon at δ 73.4, showed cross peaks, in the DQ-COSY experiment, with two diastereotopic methylene protons at δ 1.53 and 1.94, correlated to the carbon at δ 40.9, identified as the C-15 carbon. Moreover, the H-11 methine correlated in the same experiment with the H-12 methylene protons at δ 1.02 and 2.04. Heterocorrelated 2D NMR experiments (H2BC and HMBC) allowed the complete assignment of all of the ^13^C and ^1^H signals (Table 1 and Figures S6 and S7). These data were confirmed by comparison of NMR data of pure standard acquired in the same conditions (Supplementary Figures S8–S11) [14,15,16,17,18].

## 3. Discussion

In the vast scenario of microbial transformations to obtain drugs, bacteria of the genera *Arthrobacter* and *Streptomyces* have already been employed as whole cell biocatalysts in mixed cultures or in multi-step bioconversions, but more frequently alone than coupled with other microorganisms. For example, a mixture of culture broths was used to obtain the antibiotic byphenomycin-A through *Pseudomonas maltophilia* hydrolysis of an argynilserine residue of the antibiotic precursor synthesized by S. *griseorubiginosus* [19,20]. A sequential approach was used to biosynthesize 9-β-D-arabinofuranosylguanine, a powerful anti-leukemic agent, through a transglycolsilation of a 2,6-diaminopurine by *Enterobacter gergoviae* followed by a deamination reaction catalyzed by *Arthrobacter oxydans*. In this work, the possibility to exploit a coupled reaction of the strains was also investigated [21]. In addition, as concerns the biotechnological production of steroids, literature data report the single use of *A. simplex* or *S. roseochromogenes* as whole cell biocatalysts to obtain structural tailored-cut molecules. More rarely, these strains were coupled together or with other microorganisms [3,4,11]. For example, *A. simplex* was used with *Aspergillus ochraceus* in a two-step microbial transformation of 16α-hydroxycortexolone to obtain its 1-dehydro-11α-hydroxy derivative [22]. However, to the best of our knowledge, only one paper reports the contemporary use of *A. simplex* or *S. roseochromogenes* to Δ^1^-dehydrogenate and 16α-hydroxylate 9α-fluorohydroxycortisone [17]. In this study, for the first time, we explored the possibility to produce 16α-hydroxyprednisolone, an anti-inflammatory drug, starting from hydrocortisone by coupling a 1,2-dehydrogenation reaction performed by *A. simplex* with a 16α-hydroxylation by *S. roseochromogenes* [14]. The research presented above aimed to find the common conditions at which both reactions could be performed in one pot. Since the optimized conditions for *S. roseochromogenes* conversion of hydrocortisone into 16α-hydroxyhydrocortisone has already been assessed [14,15], we carried out *A. simplex* physiological studies to test the strain’s ability to convert hydrocortisone into prednisolone by using media and culture conditions already screened in the previous case. Differently from *S. roseochromogenes* that needed specific pH and temperature conditions to perform the hydroxylation of HC, *A. simplex* resulted more flexible and able to highly convert HC into PD, when grown on GYA medium in all the diverse temperature and pH conditions used (79.0–94.0%) and in only 24 h. Previous data report similar bioconversion (93.0%) of HC into PD but in longer time (120 h) when *A. simplex* was grown at 28 °C and at pH 7.0 on a complex medium containing glucose, peptone and corn step liquor, with substrate addition at 24 h [23]. Besides, growing *A. simplex* on GYA medium improved cell capability to bioconvert HC into PD more than GEM III N. The flexibility shown by *A. simplex* to highly perform the bioconversion could also be useful in the research of common conditions at which the two strains could work contemporaneously. The pH value seemed to constitute a key parameter for coupling the enzymatic activity of the two microorganisms. Our results demonstrate that pH 6.0 supported a good bioconversion rate, similar to what was previously reported in the conversion of 9α-fluorohydroxy cortisone into Δ^1^-dehydro-9α-fluoro-16α-hydroxyhydroxycortisone [17]. The possibility of each strain to transform the bioconversion product of the other microorganism has been shown in the present experimental work. To the best of our knowledge, this has not been previously tested or reported. Our results demonstrate that *A. simplex* was so flexible as to perform the 1,2-dehydrogenation reaction with high bioconversion also on 16α-hydroxyhydrocortisone, the product of *S. roseochromogenes* 16α-hydroxylation of hydrocortisone. The versatility of *S. roseochromogenes* P-450 cytochrome multi-enzymatic complex to perform, according to the growth conditions, hydroxylation or di-hydroxylation reactions in different positions of various steroidal rings is well known [5,14,15,24,25]. In this work, the microorganism also proved to perform at the same time different reactions on the same substrate, such as a 16α-hydroxylation and an 1,2 hydrogenation that transformed the prednisolone back to hydrocortisone that was then again hydroxylated at 16α- and 20-positions. In a previous study, the same strain demonstrated to be so versatile as to convert the hydrocortisone to its 16α-,20-hydroxylated products and to a 21-*N*-acetamide derivate at the same time [14,15]. In the literature, 1,2-hydrogenation reactions are described for the *S. cinereocrocratus* strain on substrates such as dehydrogriseofulvin and (-)-α-santonin [26]. Scanning electron microscopy analyses of the two microorganisms allowed us to study the strain morphology in order to point out whether it would be influenced by the steroid bioconversion process. The rod shape of *A. simplex* cells, observed since the first hours of growth, is considered typical during its exponential phase [27]. The introflections and the concave notches that were clearly visible on the bacterial membrane at 24 and 44 h aroused the hypothesis that the organic solvent used to solubilize the steroid substrate damaged the *A. simplex* cells. Similar bacterial membrane injuries, such as wrinkles, gross creases and small pores on the cell surface, were previously observed by using both transmission electron microscopy and atomic force microscopy during the dehydrogenation reaction of cortisone acetate [28,29,30]. Their presence was effectively correlated to an increased membrane instability and a loss of integrity induced by the solvents used to solubilize the substrates, but the derived enhanced membrane permeability also increased the *A. simplex* catalytic performance. To avoid cell damage and inactivation of the enzymes, aqueous two-phase systems have frequently been proposed as an alternative to perform *A. simplex* bioconversions, although they usually drive to very low yields [7,19]. Taking into consideration all these issues, the optimization of *A. simplex* bioconversion of HC into PD in the shortest possible time (in our case, in 24 h) might contribute to reduce the contact with the solvent to the bare minimum and to keep the strain cells in good conditions and active for bioconversion. The time in which *S. roseochromogenes* performed its bioconversion was longer and the SEM analyses showed a progressive and classical morphology change of the strain with increase of budding signs, cell tangling and branching, without any evident signs of cell stress. The development of these highly intricate hyphae structures is typical of the growth of this genus and it varied according to the metabolic changes of its life cycle [31,32]. These morphological changes are frequently studied in correlation to the onset of the secondary metabolism and, for example, of the antibiotic production. They have been more rarely investigated during *Streptomycetes* bioconversion processes [33,34]. Accumulation of material on the cell surface was the only sign of the progression of the microbial transformation and it resulted different from any other phenomena of entangling of branched hyphae or of fiber deposition previously reported for this genus in liquid medium suspensions [35,36]. All the detected differences between the two strain morphologies during the microbial bioconversions seemed to sustain the hypothesis that *S. roseochromogenes* cells are better at performing long-term transformations without any damage compared to *A. simplex* cells. Thus, as expected, in the two-step bioconversions, we set up shorter transformation times for *A. simplex* (24 h) and longer ones for *S. roseochromogenes* (96 h), considering the strain specific kinetic of substrate conversion. In whole cell bioconversion experiments *A. simplex* demonstrated to convert the HC to PD with percentages similar to the ones obtained in shake flasks (84.0–88.0%) while the hydroxylation capability of *S. roseochromogenes* resulted lower than the one previously reported, thus confirming the idea that the P-450 cytochrome multi-enzymatic complex works better in growing cells [14,15,22]. Data also show that at 30 °C *S. roseochromogenes* hydroxylation capability seems to be impaired if compared to the capability at 26 °C, confirming that temperature conditions deeply influenced the strain catalytic performances [14,15]. Temperature also influenced the specificity of *S. roseochromogenes* conversion of PD that was hydrogenated back to HC in higher percentage at 30 °C rather than at 26 °C. The one-step bioconversion system gave more successful results, demonstrating that coupling these two strains in their best conversion conditions helps to improve their catalytic performances and allows obtaining a 68.8 ± 0.2% maximum of 16α-OH-PD in 120 h, with low percentages of residual unconverted 16α-OH-HC and prednisolone.

## 4. Material and Methods

### 4.1. Materials

The yeast and malt extracts used in the growth media were purchased from Organotechnie (France), while all the other nutrients and salts (glucose, NH_4_SO_4_, KH_2_PO_4_, K_2_HPO_4_ and calcium acetate) were furnished by Sigma-Aldrich (St. Louis, MO, USA). Hydrocortisone and prednisolone standards were from Sigma-Aldrich (St. Louis, MO, USA), while 16α-hydroxyprednisolone standard was from Cayman chemical (Ann Arbor, MI, USA). Pure 16α-hydroxyhydrocortisone was obtained in our lab by the bioconversion of hydrocortisone in a batch growth of *S. roseochromogenes* and following reverse phase chromatography purification of the broth supernatant, as previously reported [14,15]. The *N*,*N*-dimethyl-formamide (DMF), ethanol, acetonitrile, formic acid, ethyl acetate and paraformaldehyde used for extracting the conversion products from the fermentation broth supernatants, preparing the samples and the buffers for the high pressure liquid chromatography analyses, or preparing samples for scanning electron microscope analyses were furnished by Carlo Erba (Cornaredo, Italy). Phosphate buffer saline (PBS) used for preparing samples for scanning electron microscope analyses was from Sigma-Aldrich (St. Louis, MO, USA).

### 4.2. Microorganisms and Media

The bacterial strains *Arthrobacter simplex ATCC*31652 and *Streptomyces roseochromogenes ATCC*13400 were purchased from DSMZ (Braunschweig, Germany). The strains were stored and maintained in 20% (*v*/*v*) glycerol stock solutions at −80 °C. Two media were used to test the optimal conditions of *A. simplex* growth*:* GYA medium (30 g∙L^−1^ glucose, 20 g∙L^−1^ yeast extract and 2 g∙L^−1^ ammonium sulfate) or GEM III N medium (12 g∙L^−1^ glucose, 6 g∙L^−1^ yeast extract and 30 g∙L^−1^ malt extract). GEM III N medium was also used for the growth of *S. roseochromogenes*. Both media contained 4.29 g∙L^−1^ of KH_2_PO_4_ and 17.42 g∙L^−1^ of K_2_HPO_4_ to keep the pH at 7.0 or 42.87 g∙L^−1^ of KH_2_PO_4_ and 17.42 g∙L^−1^ of K_2_HPO_4_ to keep the pH at 6.0. In all the experiments, the media were sterilized by autoclave, without glucose and K_2_HPO_4_ that were then added to the media after filtration on 0.22-μm membranes (Millipore, France). Concentrated solutions of hydrocortisone, prednisolone, 16α-hydroxyhydrocortisone or 16α-hydroxyprednisolone (180 g∙L^−1^) were prepared by dissolving in DMF to be used as substrates in the experiments.

### 4.3. Shake Flask Experiments

#### 4.3.1. *A. simplex* Shake Flask Experiments

*A. simplex* shake flask cultivations were carried out for 48 h on two different media, GYA and GEM III N, at two pH values (6.0 and 7.0) and two temperatures (26 and 30 °C) in a rotary air shaker (model Minitron, Infors, Bottmingen, Switzerland) with a speed of 200 rpm, in 1-L baffle-equipped flasks containing 200 mL of the media. Then, 0.111 mL of the HC concentrated solution (180 g∙L^−1^) was added at the beginning of the growth in order to have a final substrate concentration in shake flasks of 0.1 g∙L^−1^. Samples of broth were collected at different time points to verify the bacterial growth by measuring the absorbance at 600 nm (Spectrophotometer DU800, Beckman Coulter, Brea, CA, USA). At 24 and 48 h, aliquots of the samples (3 mL) were centrifuged at 6000 rpm for 20 min and 4 °C (Avant J-20XP, Beckman Coulter, Brea, CA, USA), and the obtained supernatants were then extracted to with ethyl acetate to determine the concentrations of the products obtained in the transformation reactions and to determine the glucose concentration.

#### 4.3.2. Shake Flask Experiments on Different Substrates

*A. simplex* and *S. roseochromogenes* shake flask experiments on different substrates were performed for 24 h on GYA medium and 96 h on GEM III N medium, respectively, all at pH 6.0 and 26 °C, as described above or in previous papers [14,15], by adding 0.1 g∙L^−1^ of hydrocortisone, 16α-hydroxyhydrocortisone or prednisolone. In this case as well, samples of broth were withdrawn to verify the bacterial growth and the bioconversion percentage, as previously described [14,15].

### 4.4. Whole Cell Experiments

A fresh wet biomass for each strain was obtained in 1-L shake flasks by growing *A. simplex* and *S. roseochromogenes* for 24 h on GYA medium and 96 h on GEM III N medium, respectively, both at pH 6.0 and 26 °C, as described above or in previous papers [14,15]. One gram of wet biomass of each strain was re-suspended singularly or together in 20 mL of a bioconversion medium made of 30.0 g∙L^−1^ glucose and phosphate buffer at pH 6.0, and then used in whole cell experiments performed in 100-mL baffled shake flasks in a rotary air shaker at 200 rpm at pH 6.0 and 26 or 30 °C, with 0.1 g∙L^−1^ of HC added at the beginning. Whole cell experiments were performed according to three different schemes (Figure 1).

#### 4.4.1. Whole Cell Experiments by Using *S. roseochromogenes* and *A. simplex* in a Sequential Mode

In Scheme A, the two reactions were performed by the two strain cells in a sequential mode, first *S. roseochromogenes* cells were used to perform a 96-h bioconversion, then the supernatant was recovered by centrifugation at 6000 rpm at 4 °C for 20 min (Avant J-20XP, Beckman Coulter, Brea, CA, USA) and then used as substrate for a 24-h bioconversion by *A. simplex* cells (total time 120 h).

#### 4.4.2. Whole Cell Experiments by Using *A. simplex* and *S. roseochromogenes* in a Sequential Mode

In Scheme B, the sequence of the reactions was inverted and first *A. simplex* cells were used to perform a 24-h bioconversion, and then the supernatant was recovered by centrifugation as described above and used as substrate for a 96-h bioconversion by *S. roseochromogenes* (total time 120 h).

#### 4.4.3. Whole Cell Experiments by Coupling *A. simplex* and *S. roseochromogenes*

In Scheme C, the two reactions were simultaneously performed by coupling the cells of the two strains (total time 120 h). Small volumes (2 mL) of the reaction were withdrawn at different time points, centrifuged at 6000 rpm at 4 °C for 20 min (Avant J-20XP, Beckman Coulter, Brea, CA, USA), and the supernatants were extracted to determine the concentrations of the substrate and the products obtained in the transformation reactions or used to determine the glucose concentration. Three 20-mL bioconversion whole cell experiments were also performed similarly by coupling the cells of the two strains at pH 6.0 and at 26 °C, with 0.1 g∙L^−1^ of HC as initial substrate concentration, to produce enough 16α-OH-PD to be then purified and characterized by NMR.

### 4.5. Glucose Determination

The supernatants of samples from shake flask cultures or from whole cell experiments (0.5 mL) were ultrafiltered/diafiltered on 3-kDa centrifugal filter devices (Centricon, Amicon, USA) at 12,000 rpm and 4 °C (Centifuge Z216MK, Hermle Labortechnik, Wehingen, Germany), and the permeate volumes were analyzed by high performance anion exchange chromatography with pulsed amperometric detection (HPAE-PAD) (model ICS-3000, Dionex, Sunnyvale, CA, USA) to determine the concentration of glucose at different time points. Analyses were performed by using a Carbopac PA1 column (Dionex, Sunnyvale, CA, USA) at 25 °C, eluting in isocratic conditions with 237 mM NaOH at a flow rate of 1 mL∙min^−1^ as previously reported [37].

### 4.6. Steroid Extraction and HPLC Analyses

Aliquots (1 mL) of the supernatant samples of the shake flasks or of the whole cell experiments were extracted three times consecutively by using an equal volume of ethyl acetate (total volume of the extracted sample = 3 mL) and analyzed by HPLC (Beckman Coulture, Brea, CA, USA), according to procedures previously reported [14,15]. Briefly, 1 mL of the extracted samples were evaporated in a vacuum centrifuge (Centrifuge 5415 R, Eppendorf, Ocala, FL, USA) and then re-suspended in 1 mL of ethanol. Analyses were performed by using an Allure Biphenyl column (Resteck, 250 × 4.6 mm, 5 μm, 60 A°), injecting 10 μL of sample, eluting with a AcN/H_2_0 (30/70 *v*/*v*) buffer, containing 0.1% of formic acid, at 0.8 mL·min^−1^, at 50 °C, detecting at 254 nm. Peak quantification was performed by building calibration curves of the standards or of the purified steroids, in the concentration range from 0.0025 to 0.5 g L^−1^. The peak concentrations obtained from HPLC analyses were used to calculate the conversion of HC, or of the other substrates, to the different products, namely PD, 16α-OH-HC, 20-OH-HC and 16α-OH-PD, in terms of molar concentration percentage ratio, according to the following equation:

%product = [product (mol∙L^−1^)/substrate_initial_ (mol∙L^−1^)] × 100.

### 4.7. Steroid Purification by Preparative Chromatography

The 16α-OH-PD obtained in whole cell experiments by coupling the two strains was purified according to a chromatographic method previously reported, to be then characterized by NMR [15]. Briefly, supernatant samples of the HC bioconversion were loaded (10 mL) on a reverse phase column (RPC Resource, GE Healthcare, Milan, Italy) connected to a chromatographic system (ÄKTA explorer 100, GE Healthcare, Milan, Italy), equipped with two piston pumps, an UV detector, a pH meter, a conductivity cell and a fraction collector, and controlled by software (Unicorn 5.0, GE Healthcare, Milan, Italy). Samples were eluted with the following profile: 95% A and 10% B for 5 column volumes (cv), 85% A and 15% B in 5 cv, 80% A and 20% B in 5 cv and then from 75 to 5% A and from 25 to 95% B in 3 cv [A = 0.1% trifluoracetic acid (*v*/*v*) in deionized water; B = 0.1% trifluoracetic acid (*v*/*v*) in acetonitrile]. A washing step of 95% buffer B and an equilibration one of 5% buffer B for 5 column volumes each were performed at the end of each run. Detection was performed by monitoring the absorbance at 254 nm. 16α-OH-HC produced by *S. roseochromogenes* was purified with the previously reported method [14,15] and used in this study as a different substrate in shake flask experiments. Fractions containing the peaks were pooled together peak by peak, and then they were evaporated by a vacuum centrifuge (Concentrator 580, Eppendorf, Hamburg, USA) and analyzed by HPLC, as reported above, to determine the steroid content and purity. The purity of the 16α-OH-PD was calculated based on the HPLC analyses by dividing the concentration of the 16α-OH-PD peak to the sum of the concentrations of all the steroidal peaks in the chromatogram, according to the following formula:

%16α-OH-PD purity = [16α-OH-PD (mol∙L^−1^)/Σ steroids (mol∙L^−1^)] × 100.

### 4.8. NMR Analyses

^1^H, ^13^C, DQF-COSY and HSQC experiments were performed to characterize the purified 16α-OH-PD. NMR spectra were recorded at 25 °C on a NMR Varian Unity Inova 500 MHz Spectrometer and a Varian Mercury Plus 300 Fourier transform NMR. CD_3_OD was used as the internal lock. Chemical shifts are reported in δ (ppm) and referenced to the residual solvent signal; J (coupling constant) are given in Hz. Standard pulse sequences and phase cycling from Varian library were used for ^1^H, ^13^C, DQF-COSY and HSQC, experiments. ^1^H NMR spectra were acquired over a spectral window from 14 to −2 ppm, with 1.0 s relaxation delay, 1.70 s acquisition time (AQ) and 90° pulse width = 13.8 μs. The initial matrix was zero-filled to 64 K. ^13^C NMR spectra were recorded in ^1^H broadband decoupling mode, over a spectral window from 235 to −15 ppm, 1.5 s relaxation delay, 90° pulse width = 9.50 μs and AQ = 0.9 s. The number of scans for both ^1^H and ^13^C NMR experiments were chosen depending on the concentration of the samples. With regards to the homonuclear and heteronuclear 2D-NMR experiments, data points, number of scans and increments were adjusted according to the sample concentrations. Double quantum filtered correlation spectroscopy (DQF-COSY) spectrum was recorded with gradient enhanced sequence at spectral widths of 3000 Hz in both f2 and f1 domains; the relaxation delays were of 1.0 s. Proton-detected heteronuclear correlations were measured. Heteronuclear single-quantum coherence (HSQC) experiments (optimized for ^1^J_(H,C)_ = 140 Hz) were performed in the phase sensitive mode with field gradient. The spectral width was 12,000 Hz in f1 (^13^C) and 3000 Hz in f2 (1H) and 1.0 s of relaxation delay; the matrix of 1 k × 1 k data points was zero-filled to give a final matrix of 2k × 2k points. Heteronuclear 2 bond correlation (H2BC) spectra were obtained with T = 30.0 ms and a relaxation delay of 1.0 s; the third order low-pass filter was set for 130 < ^1^J_(C,H)_ < 165 Hz. Heteronuclear multiple bond coherence (HMBC) experiment (optimized for ^1^J_(H,C)_ = 8 Hz) was performed in the absolute value mode with field gradient; typically, ^1^H–^13^C gHMBC were acquired with spectral width of 18,000 Hz in f1 (^13^C) and 3000 Hz in f2 (^1^H) and 1.0 s of relaxation delay; the matrix of 1k × 1k data points was zero-filled to give a final matrix of 4k × 4k points.

### 4.9. Scanning Electron Microscope Analyses

Samples of the two strains from the shake flask experiments were analyzed by scanning electron microscopy (SEM). They were first washed in PBS, then fixed in a 4.0% paraformaldehyde solution in PBS, dehydrated for 5 min with increasing ethanol percentage solutions (from 30.0% to 100.0% ethanol in water), treated in Critical Point Dryer (EMITECH K850), sputter coated with platinum–palladium at 77 mAmps for 120 s (Denton Vacuum DESKV) and observed with field-emission scanning electron microscope (Supra 40 Zeiss, EHT = 5.00 kV, WD = 22 mm, detector inlens). Cell dimensions were measured using Smart-Sem software (Zeiss).

### 4.10. Data and Statistical Analyses

All the results and the calculated values that are reported in the text, tables and figures are average values of three independent experiments calculated with their standard deviations by a Microsoft Office Excel 2007 program (Microsoft, Redmond, DC, USA). Statistical comparison between groups of data, for example between the results of the different shake flask runs, were determined by the t-student test by the Microsoft Office Excel 2007 program as well, and the data were considered significantly different if p values were lower than 0.05 or 0.01.

## 5. Conclusions

For the first time, 16α-hydroxyprednisolone was obtained by the bioconversion of hydrocortisone in a 20-mL scale system by coupling *A. simplex* and *S. roseochromogenes* whole cells. The two strains were able to work together, achieving a sound bioconversion efficiency, specificity and high transformation percentages. All these results are the first step for further investigations to achieve a scalable process.

## Figures and Tables

**Table 1:** 1D and 2D NMR data of 16α-OH-prednisolone in CD_3_OD.

Position	δ_C_	Type	δ_H_ (*J* in Hz)	COSY	HMBC (H→C)	H2BC (H→C)
1	159.9	CH	7.45 *d* (9.9)	2	3, 5, 6, 9, 10	2
2	127.9	CH	6.25 *d* (9.9)	1	5, 10	1
3	188.9	C	-	-	-	-
4	122.6	CH	6.00 *s*	-	2, 6, 10	-
5	174.6	C	-	-	-	-
6	33.1	CH_2_	2.37 *dd* (9.3, 5.1)	7	4, 5, 7, 8, 10	7
			2.64 *ddd* (9.3, 4.5, 1.0)	7	4, 5, 7, 8, 10	7
7	35.5	CH_2_	1.17 *m*	6	6, 10, 14	6, 8
			2.09 *m*	6, 8	6, 10, 14	6, 8
8	32.3	CH	2.13 *ov*	9	10, 14	9, 7, 14
9	57.2	CH	1.03 *dd* (11.0, 3.5)	8, 11	7, 9, 10, 11, 14, 19	8, 11
10	46.1	C	-	-	-	-
11	70.6	CH	4.38 *m*	9, 12	9, 12	9, 12
12	40.9	CH_2_	1.56 *m*	11	11	11
			2.03 *m*	11	11	11
13	48.9	C	-	-	-	-
14	51.6	CH	1.92 *ov*	15	9, 15, 17	9, 15
15	35.2	CH_2_	1.52 *ov*	14, 16	14, 16, 17	14, 16
			1.93 *m*	16	14, 16, 17	14, 16
16	73.5	CH_2_	4.92 *m*	15	14, 20	15
17	89.3	C	-	-	-	-
18	17.8	CH_3_	0.99 *s*	-	12, 13, 14, 17	-
19	21.6	CH_3_	1.49 *s*	-	1, 5, 9, 10	-
20	213.0	C	-	-	-	-
21	68.2	CH_2_	4.26 *d*	21	20	-
			4.62 *d*		20	-

*d*, doublet; *m*, multiplet; *ov*, overlapped; *s*, singlet; *dt*, double of triplets; *dd*, double of doublets.

## Supplementary Materials

The following are available online. Table S1. Shake flask experiments of *A. simplex*: initial and final glucose concentrations on GYA or GEM III N media, at pH 6.0 or 7.0, at 26 °C or at 30 °C., Figure S1. Growth curves of *A. simplex* and *S. roseochromogenes* in shake flask experiments on GYA and GEM III N media, respectively, at pH 6.0 and at 26 °C with initial addition of 0.1 g·L−1 of 16α-OH-HC and of PD, respectively., Figure S2. Overlaid HPLC chromatograms of the 16α-OH-PD standard (blue line) and of the sample of 0.1 g·L^−1^ HC bioconversion (black line) obtained by coupling *A. simplex* and *S. roseochromogenes* in whole cell experiments at pH 6.0 and at 26 °C (A). HPLC chromatogram of the purified 16α-OH-PD (B). All the peaks are indicated by the arrows., Figure S3. ^1^H NMR of 16α-OH prednisolone obtained by bioconversion of HC by coupling *A. simplex* and *S. roseochromogenes.*, Figure S4. 13C NMR of 16α-OH prednisolone obtained by bioconversion of HC by coupling *A. simplex* and *S. roseochromogenes*., Figure S5. HSQC of 16α-OH prednisolone obtained by bioconversion of HC by coupling *A. simplex* and *S. roseochromogenes.*, Figure S6. H2BC of 16α-OH prednisolone obtained by bioconversion of HC by coupling *A. simplex* and *S. roseochromogenes.*, Figure S7. HMBC of 16α-OH prednisolone obtained by bioconversion of HC by coupling *A. simplex* and *S. roseochromogenes.*, Figure S8. ^1^H NMR of prednisolone standard., Figure S9. ^13^C NMR of prednisolone standard., Figure S10. ^1^H NMR of hydrocortisone standard., Figure S11. ^13^C NMR of hydrocortisone standard.
