# Supplementary material for: Biotechnological Transformation of Hydrocortisone into 16α-Hydroxyprednisolone by Coupling Arthrobacter simplex and Streptomyces roseochromogenes
Source: Molecules. 2020 Oct 23;25(21):4912. doi: 10.3390/molecules25214912 (PMC7660607; doi:10.3390/molecules25214912)
Supplement: Supplementary file 1 [file molecules-25-04912-s001.pdf]

molecules

MDPI

Supplementary Information

Biotechnological Transformation of Hydrocortisone into 16α-Hydroxyprednisolone by Coupling *Arthrobacter simplex* and *Streptomyces roseochromogenes*

Odile Francesca Restaino [1,*], Simona Barbuto Ferraiuolo [1], Addolorata Perna [1], Marcella Cammarota [1], Maria Giovanna Borzacchiello [1], Antonio Fiorentino [2] and Chiara Schiraldi [1]

1 Department of Experimental Medicine, Section of Biotechnology and Molecular Biology, University of Campania "Luigi Vanvitelli", Via De Crecchio 7, 80138 Naples, Italy; simona.barbutoferraiuolo@unicampania.it (S.B.F.); dolores.90@libero.it (A.P.); marcella.cammarota@unicampania.it (M.C.); mariagiovanna.borzacchiello@unicampania.it (M.G.B.); chiara.schiraldi@unicampania.it (C.S.)

2 Department of Environmental, Biological and Pharmaceutical Sciences and Technologies, University of Campania "Luigi Vanvitelli", Via Vivaldi 43, 81100 Caserta, Italy; antonio.fiorentino@unicampania.it

* Correspondence: odilefrancesca.restaino@unicampania.it; Tel.: +39-81-5667666. Fax: +39-81-5667546

Table S1. Shake flask experiments of *A. simplex*: initial and final glucose concentrations on GYA or GEM III N media, at pH 6.0 or 7.0, at 26 °C or at 30 °C.

	26 °C		30 °C	
**Glucose (g/L)**	**0 h**	**48 h**	**0 h**	**48 h**
**GYA pH 6**	30.0	8.1	30.0	4.2
**GYA pH 7**	30.1	8.2	30.1	6.0
**GEM III N pH 6**	12.0	3.6	12.1	1.7
**GEM III N pH 7**	12.0	3.8	12.1	1.3

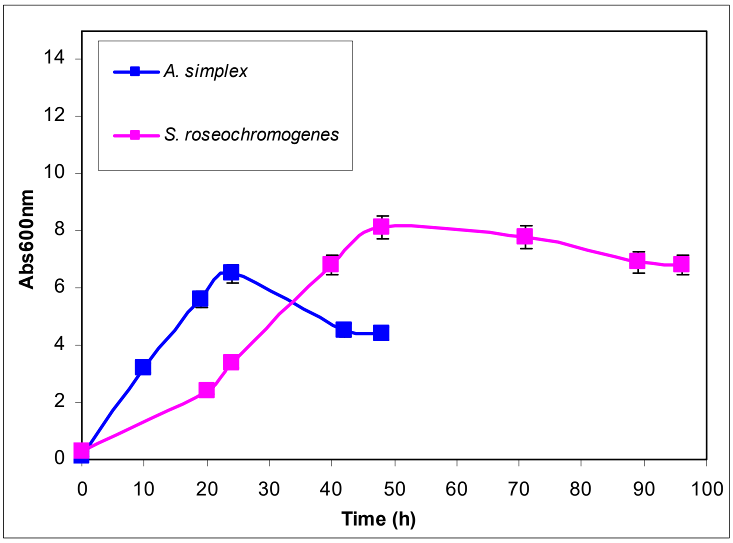

Figure S1. Growth curves of *A. simplex* and *S. roseochromogenes* in shake flask experiments on GYA and GEM III N media, respectively, at pH 6.0 and at 26 °C with initial addition of 0.1 g·L^{-1} of 16α-OH-HC and of PD, respectively.

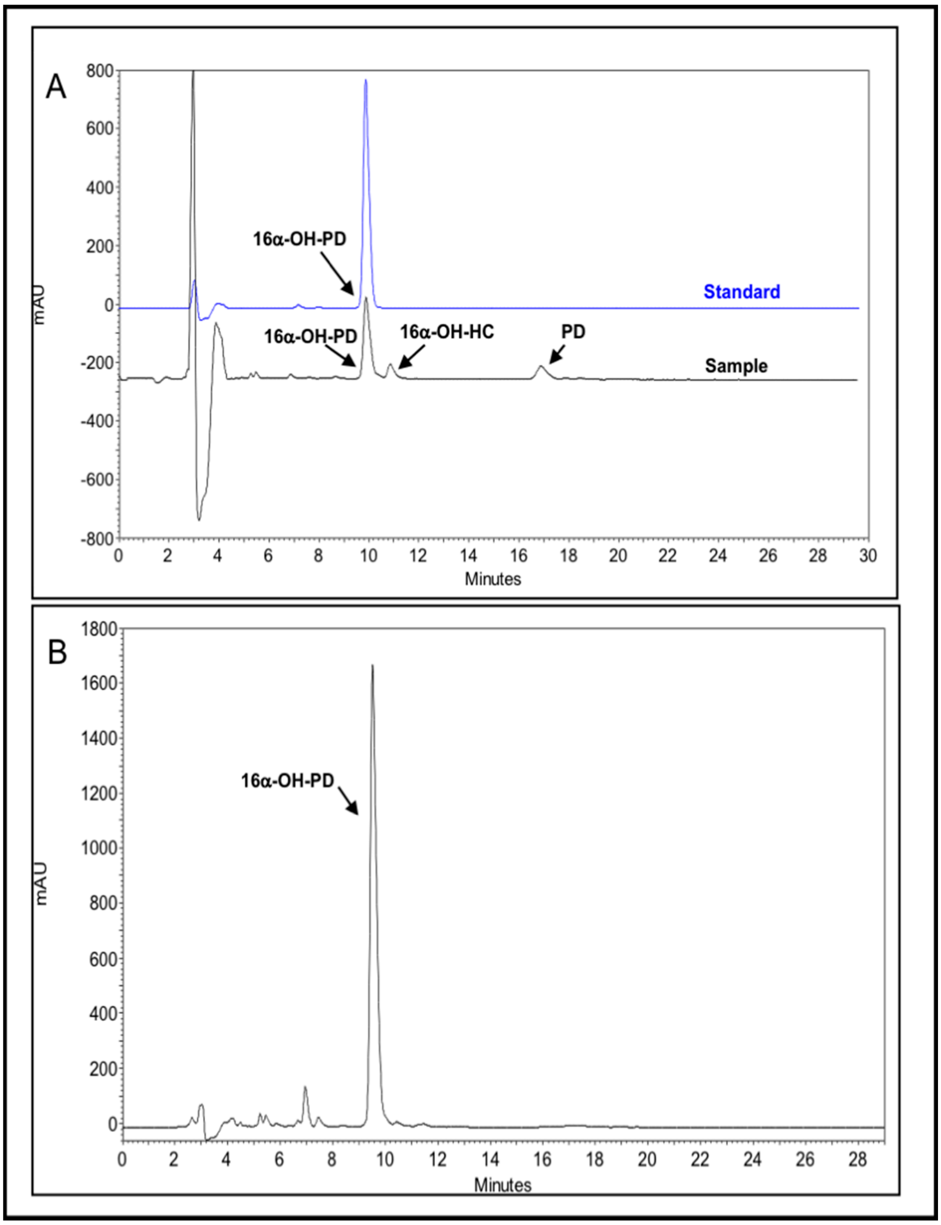

Figure S2. Overlaid HPLC chromatograms of the 16α-OH-PD standard (blue line) and of the sample of 0.1 g·L^{-1} HC bioconversion (black line) obtained by coupling *A. simplex* and *S. roseochromogenes* in whole cell experiments at pH 6.0 and at 26 °C (A). HPLC chromatogram of the purified 16α-OH-PD (B). All the peaks are indicated by the arrows.

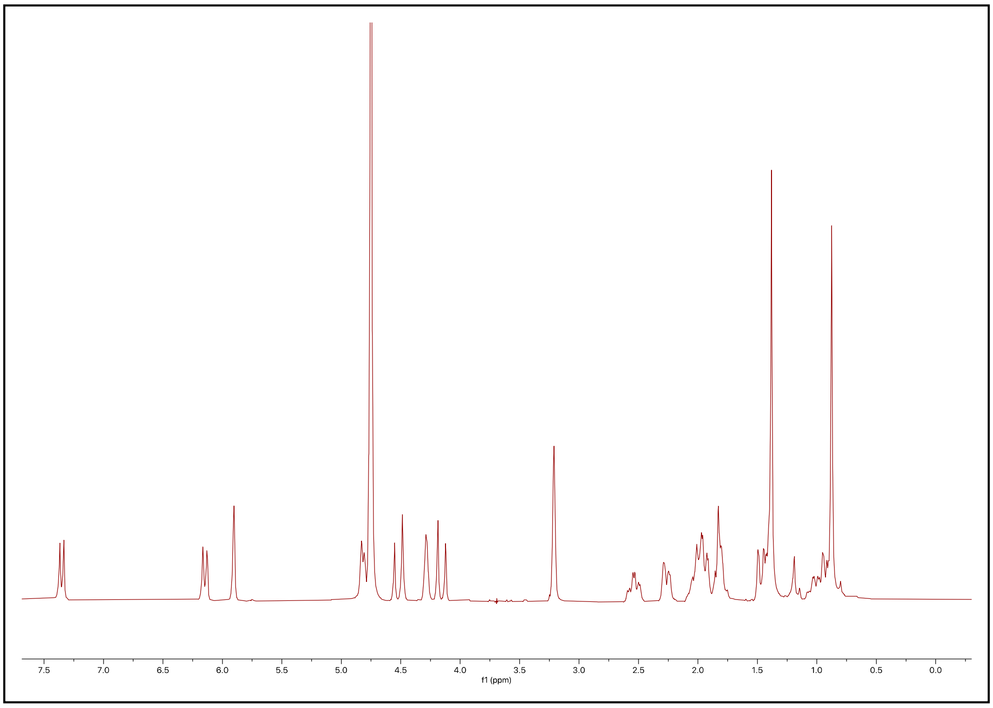

Figure S3. ^{1}H NMR of 16α-OH prednisolone obtained by bioconversion of HC by coupling *A. simplex* and *S. roseochromogenes***.**

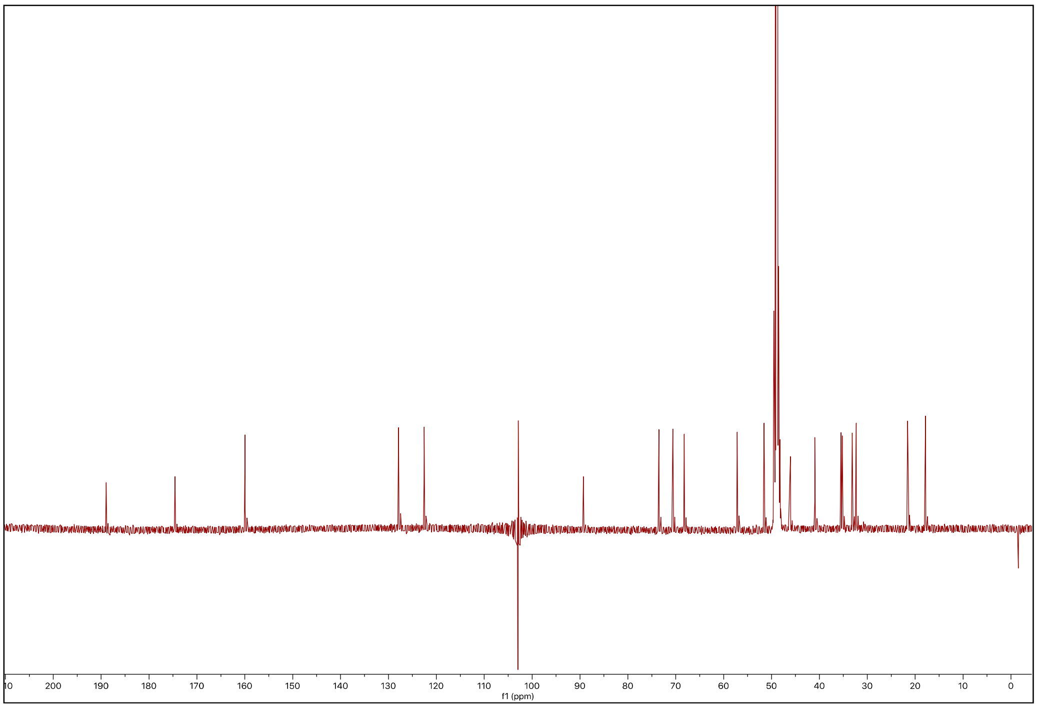

Figure S4. ^{13}C NMR of 16α-OH prednisolone obtained by bioconversion of HC by coupling *A. simplex* and *S. roseochromogenes.*

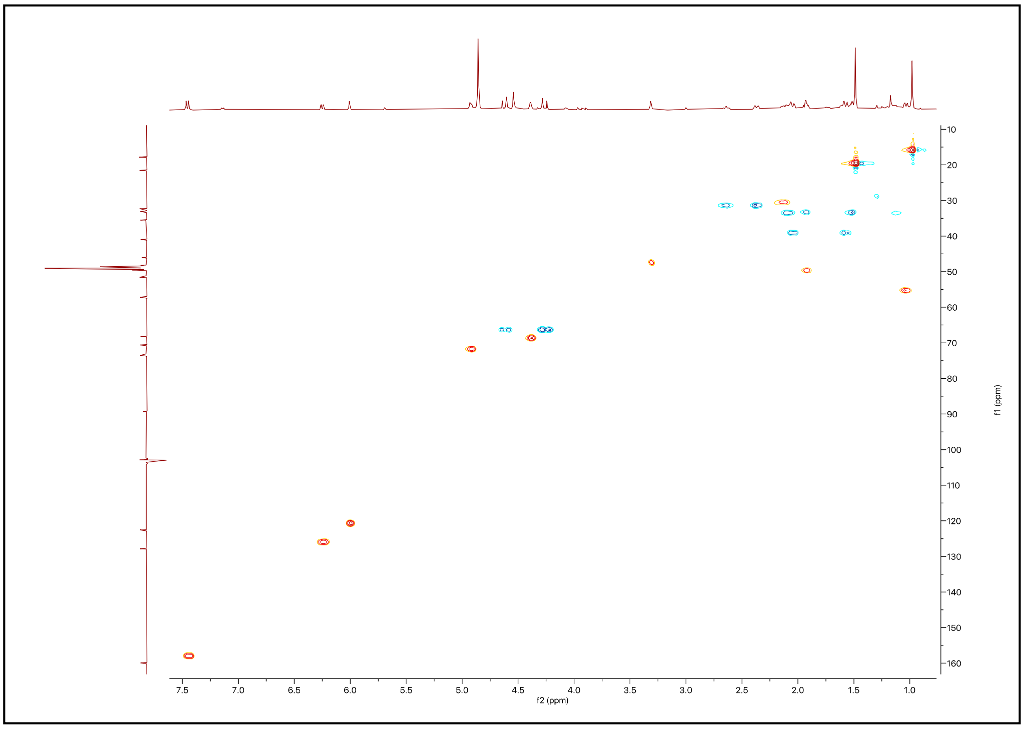

Figure S5. HSQC of 16α-OH prednisolone obtained by bioconversion of HC by coupling *A. simplex* and *S. roseochromogenes.*

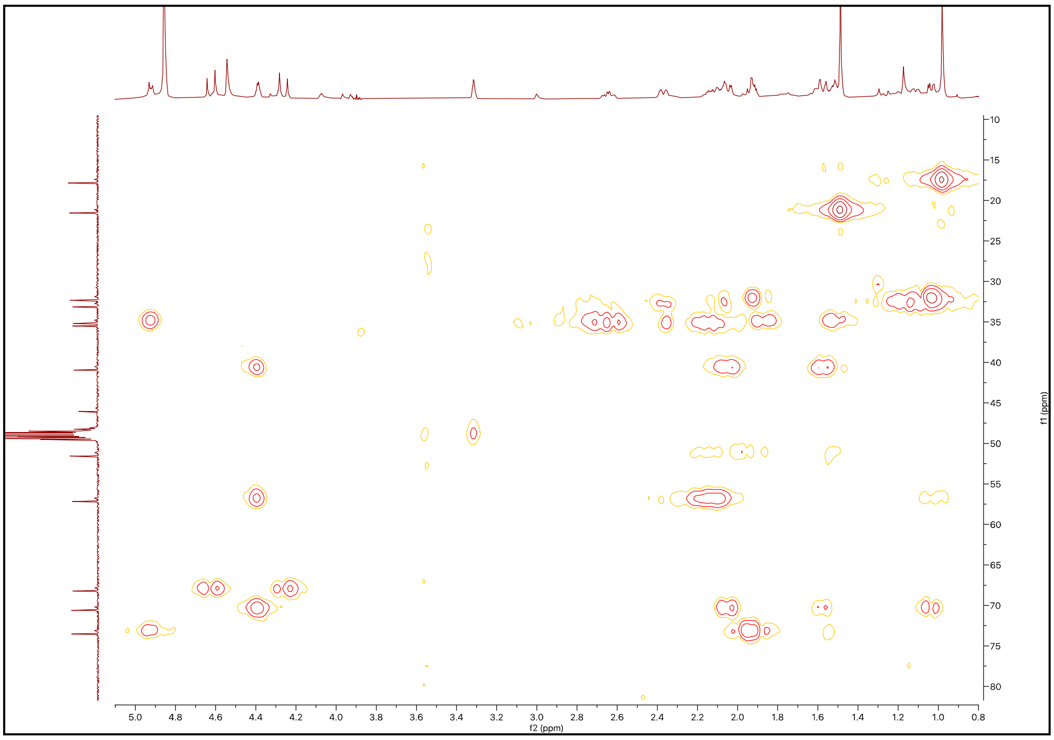

Figure S6. H2BC of 16α-OH prednisolone obtained by bioconversion of HC by coupling *A. simplex* and *S. roseochromogenes.*

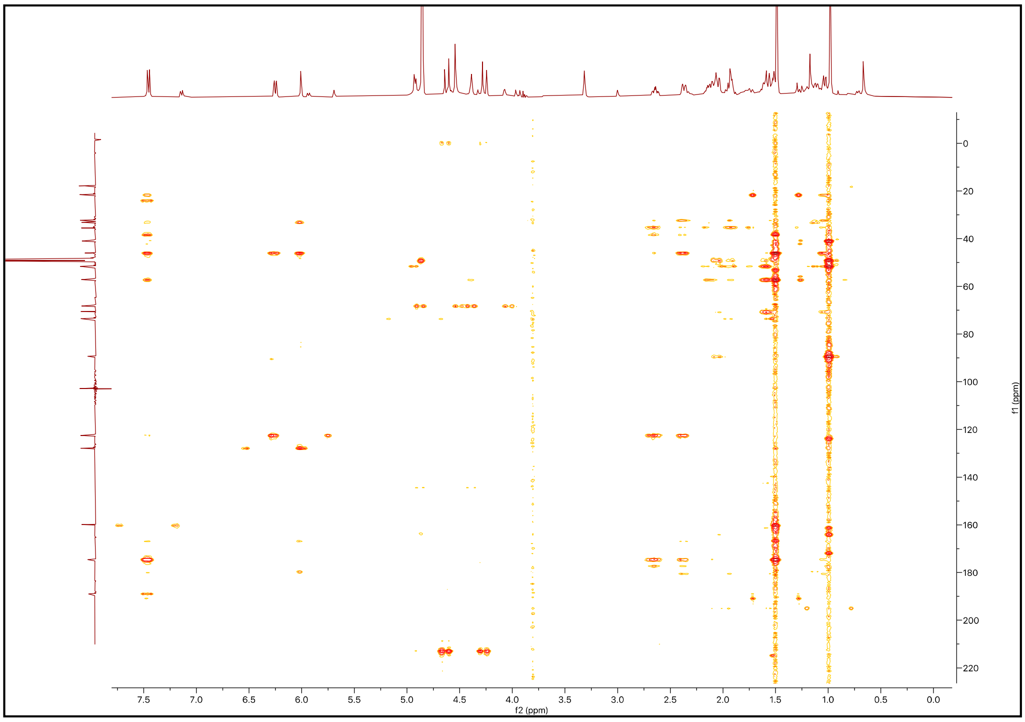

Figure S7. HMBC of 16α-OH prednisolone obtained by bioconversion of HC by coupling *A. simplex* and *S. roseochromogenes.*

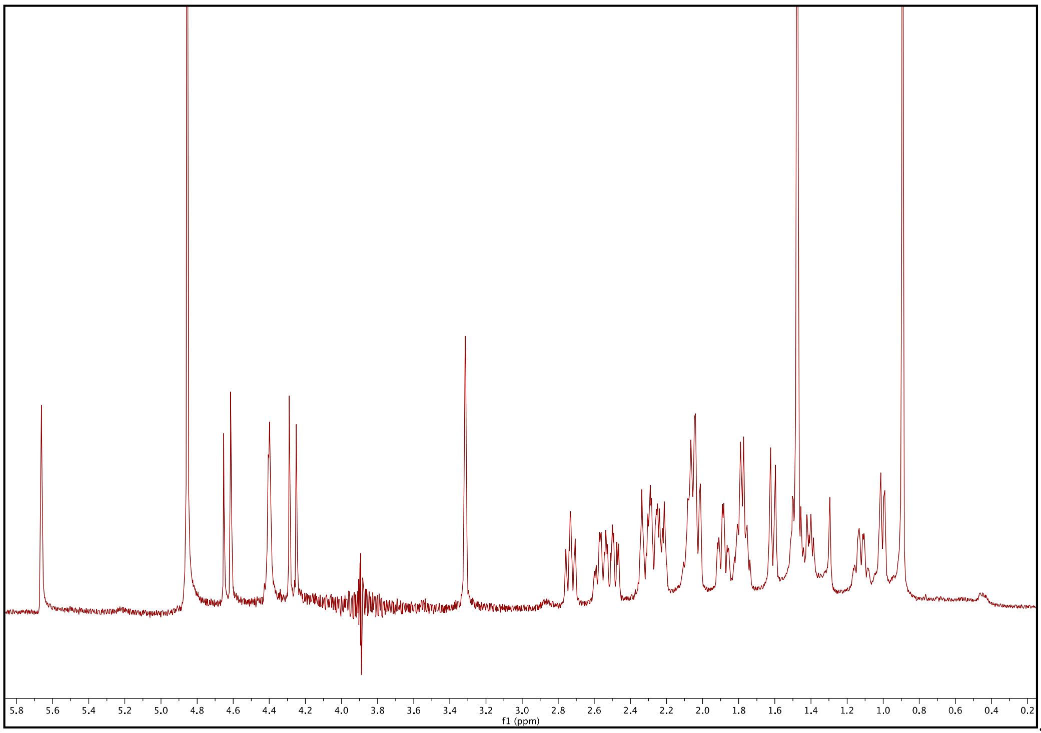

Figure S8. ^{1}H NMR of prednisolone standard.

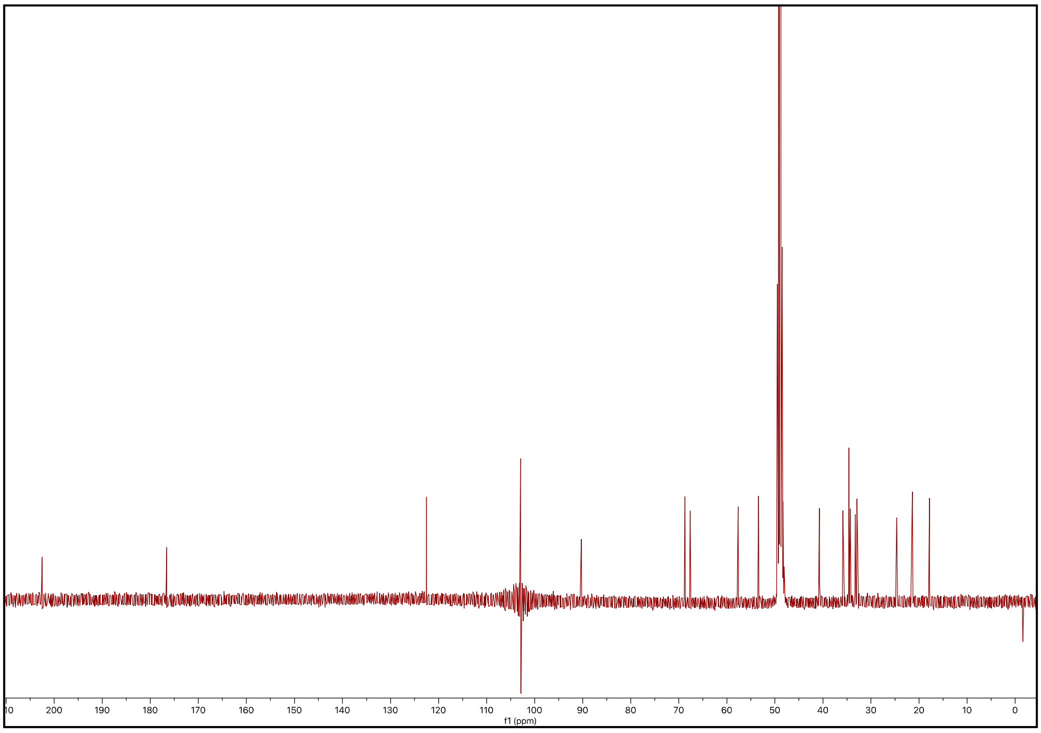

Figure S9. ^{13}C NMR of prednisolone standard.

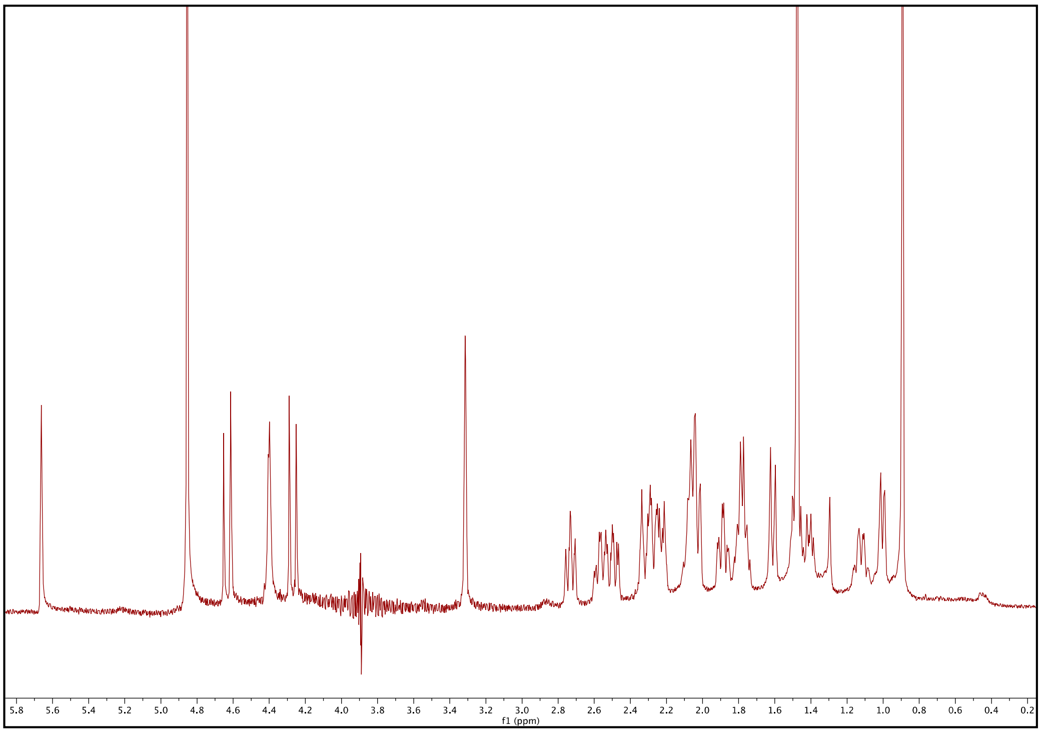

Figure S10. ^{1}H NMR of hydrocortisone standard.

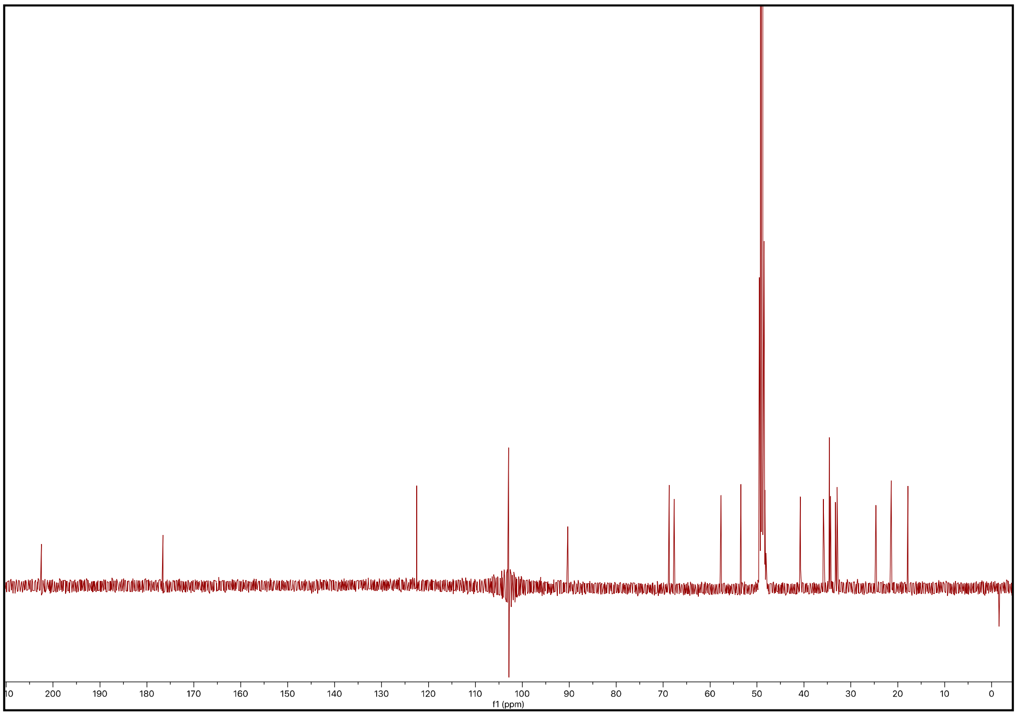

Figure S11. ^{13}C NMR of hydrocortisone standard.
